# Classification of SD-OCT Volumes Using Local Binary Patterns: Experimental Validation for DME Detection

**DOI:** 10.1155/2016/3298606

**Published:** 2016-07-31

**Authors:** Guillaume Lemaître, Mojdeh Rastgoo, Joan Massich, Carol Y. Cheung, Tien Y. Wong, Ecosse Lamoureux, Dan Milea, Fabrice Mériaudeau, Désiré Sidibé

**Affiliations:** ^1^LE2I UMR6306, CNRS, Arts et Métiers, Université Bourgogne Franche-Comté, 12 rue de la Fonderie, 71200 Le Creusot, France; ^2^Department of Ophthalmology and Visual Sciences, The Chinese University of Hong Kong, Hong Kong Eye Hospital, 147K Argyle Street, Kowloon, Hong Kong; ^3^Singapore Eye Research Institute, Singapore National Eye Center, Singapore; ^4^Electrical & Electronic Engineering Department, Center for Intelligent Signal and Imaging Research (CISIR), Universiti Teknologi Petronas, Tronoh, 32610 Seri Iskandar, Perak, Malaysia

## Abstract

This paper addresses the problem of automatic classification of Spectral Domain OCT (SD-OCT) data for automatic identification of patients with DME versus normal subjects. Optical Coherence Tomography (OCT) has been a valuable diagnostic tool for DME, which is among the most common causes of irreversible vision loss in individuals with diabetes. Here, a classification framework with five distinctive steps is proposed and we present an extensive study of each step. Our method considers combination of various preprocessing steps in conjunction with Local Binary Patterns (LBP) features and different mapping strategies. Using linear and nonlinear classifiers, we tested the developed framework on a balanced cohort of 32 patients. Experimental results show that the proposed method outperforms the previous studies by achieving a Sensitivity (SE) and a Specificity (SP) of 81.2% and 93.7%, respectively. Our study concludes that the 3D features and high-level representation of 2D features using patches achieve the best results. However, the effects of preprocessing are inconsistent with different classifiers and feature configurations.

## 1. Introduction

Eye diseases such as Diabetic Retinopathy (DR) and Diabetic Macular Edema (DME) are the most common causes of irreversible vision loss in individuals with diabetes. Just in United States alone, health care and associated costs related to eye diseases are estimated at almost $500 M [1]. Moreover, the prevalent cases of DR are expected to grow exponentially affecting over 300 M people worldwide by 2025 [2]. Given this scenario, early detection and treatment of DR and DME play a major role in preventing adverse effects such as blindness. DME is characterized as an increase in retinal thickness within 1-disk diameter of the fovea center with or without hard exudates and sometimes associated with cysts [3]. Fundus images which have proven to be very useful in revealing most of the eye pathologies [4, 5] are not as good as OCT images which provide information about cross-sectional retinal morphology [6].

**Figure 1:**
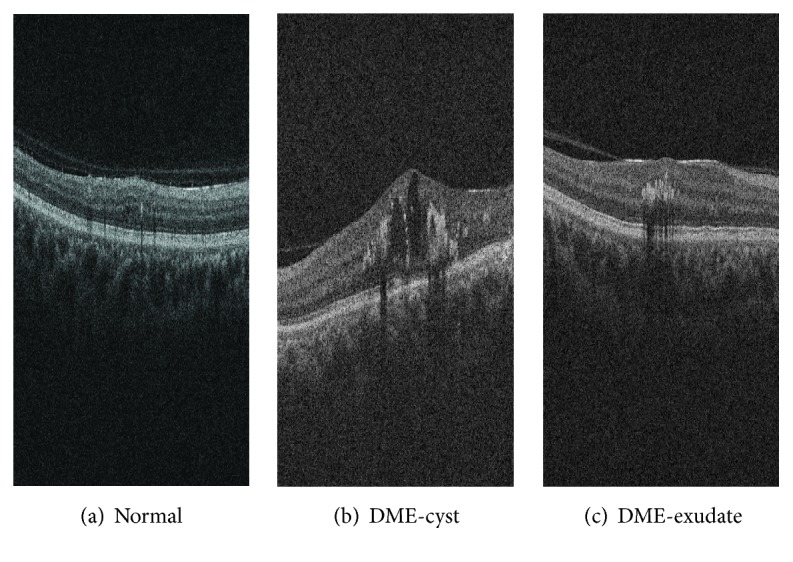
Example of SD-OCT images for normal (a) and DME patients (b)-(c) with cyst and exudate, respectively.

Many of the previous works on OCT image analysis have focused on the problem of retinal layers segmentation, which is a necessary step for retinal thickness measurements [7, 8]. However, few have addressed the specific problem of DME and its associated features detection from OCT images.  Figure 1 shows one normal B-scan and two abnormal B-scans.

A summary of the existing work can be found in Table 1. Srinivasan et al. [9] proposed a classification method to distinguish DME, Age-Related Macular Degeneration (AMD), and normal SD-OCT volumes. The OCT images are preprocessed by reducing the speckle noise by enhancing the sparsity in a transform-domain and flattening the retinal curvature to reduce the interpatient variations. Then, Histograms of Oriented Gradients (HOG) are extracted for each slice of a volume and linear Support Vector Machine (SVM) is used for classification. On a dataset of 45 patients equally subdivided into the three aforementioned classes, this method leads to a correct classification rate of 100%, 100%, and 86.67% for normal, DME, and AMD patients, respectively. The images that have been used in their paper are publicly available but are already preprocessed (i.e., denoised), have different sizes for the OCT volumes, and do not offer a huge variability in terms of DME lesions, and some of them, without specifying which, have been excluded for the training phase; all these reasons prevent us from using this dataset to benchmark our work.

Venhuizen et al. proposed a method for OCT images classification using the Bag-of-Words (BoW) model [10]. The method starts with the detection and selection of key points in each individual B-scan, by keeping the most salient points corresponding to the top 3% of the vertical gradient values. Then, a texton of size 9 × 9 pixels is extracted around each key point, and Principal Component Analysis (PCA) is applied to reduce the dimension of every texton to get a feature vector of size 9. All extracted feature vectors are used to create a codebook using *k*-means clustering. Then, each OCT volume is represented in terms of this codebook and is characterized as a histogram that captures the codebook occurrences. These histograms are used as feature vector to train a Random Forest (RF) with a maximum of 100 trees. The method was used to classify OCT volumes between AMD and normal cases and achieved an Area Under the Curve (AUC) of 0.984 with a dataset of 384 OCT volumes.

Liu et al. proposed a methodology for detecting macular pathology in OCT images using LBP and gradient information as attributes [11]. The method starts by aligning and flattening the images and creating a 3-level multiscale spatial pyramid. The edge and LBP histograms are then extracted from each block of every level of the pyramid. All the obtained histograms are concatenated into a global descriptor whose dimensions are reduced using PCA. Finally a SVM with a Radial Basis Function (RBF) kernel is used as classifier. The method achieved good results in detection OCT scan containing different pathologies such as DME or AMD, with an AUC of 0.93 using a dataset of 326 OCT scans.

Lemaitre et al. [12] proposed using 2D and 3D LBP features extracted from denoised volumes and dictionary learning using the BoW models [13]. In the proposed method all the dictionaries are learned with the same size of “visual words” (*k* = 32) and final descriptors are classified using RF classifier.

The work described in this paper is an extension of our previous work [12]. In this research, beside the comparison of 2D and 3D features, we explore different possible representations of the features and different preprocessing steps for OCT data (i.e., aligning, flattening, and denoising). We also compare the performances of different classifiers.

This paper is organized as follows: the proposed framework is explained in Section 2, while the experiments and results are discussed through Sections 3 and 4. Finally, the conclusion and avenue for future directions are drawn in Section 5.

## 2. Materials and Methods

**Figure 2:**
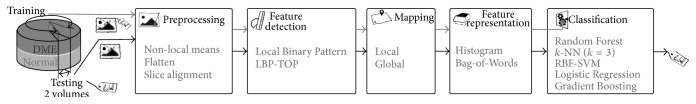
Our proposed classification pipeline.

The proposed method, as well as its experimental setup, for OCT volume classification is outlined in Figure 2. The methodology is formulated as a standard classification procedure which consists of five steps. First, the OCT volumes are preprocessed as presented in detail in Section 2.1. Then, LBP and LBP-TOP features are detected, mapped, and represented as discussed in depth in Sections 2.2, 2.3, and 2.4, respectively. Finally, the classification step is presented in Section 2.5.

### 2.1. Image Preprocessing

This section describes the set of preprocessing techniques which aim at enhancing the OCT volume. The influences of these preprocessing methods and their possible combinations are extensively studied in Section 3.

#### 2.1.1. Non-Local Means (NLM)

**Figure 3:**
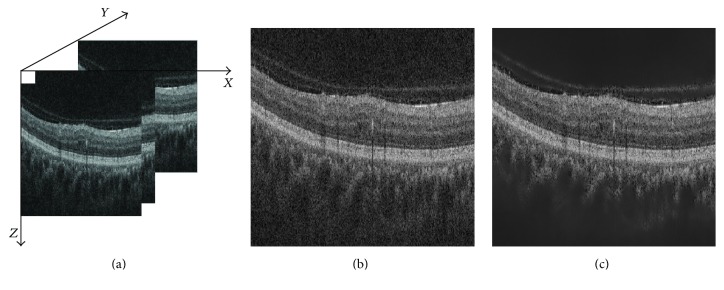
OCT: (a) organization of the OCT data, (b) original image, and (c) NLM filtering. Note that the images have been negated for visualization purposes.

OCT images suffer from speckle noise, like other image modalities such as Ultrasound (US) [14]. The OCT volumes are enhanced by denoising each B-scan (i.e., each (*x*-*z*) slice) using the NLM [15], as shown in Figure 3. NLM has been successfully applied to US images to reduce speckle noise and outperforms other common denoising methods [16]. NLM filtering preserves fine structures as well as flat zones, by using all the possible self-predictions that the image can provide rather than local or frequency filters such as Gaussian, anisotropic, or Wiener filters [15].

#### 2.1.2. Flattening

Textural descriptors characterize spatial arrangement of intensities. However, the OCT scans suffer from large type of variations: inclination angles, positioning, and natural curvature of the retina [11]. Therefore, these variations have to be taken into account to ensure a consistent characterization of the tissue disposition, regardless of the location in the retina. This invariance can be achieved in different manners: (i) using a rotation invariant descriptor (cf.  Section 2.2) or (ii) unfolding the curvature of the retina. This latter correction is known as image flattening which theoretically consists of two distinct steps: (i) estimate and fit the curvature of the Retinal Pigment Epithelium (RPE) and (ii) warp the OCT volume such that the RPE becomes flat.

**Figure 4:**
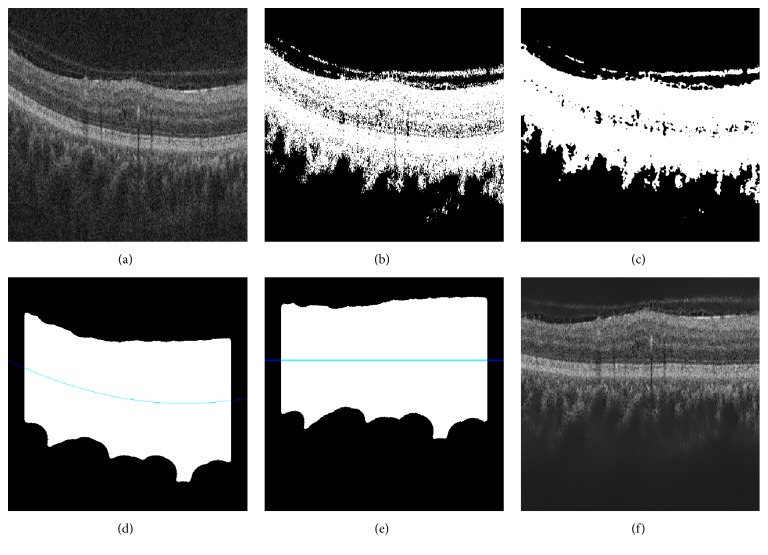
Flattening procedure: (a) original image, (b) thresholding, (c) median filtering, (d) curve fitting, (e) warping, and (f) flatten image.

Our correction is similar to the one of Liu et al. [11]: each B-scan is thresholded using Otsu's method followed by a median filtering to detect the different retina layers (see Figures 4(c) and 4(d)). Then, a morphological closing and opening is applied to fill the holes and the resulting area is fitted using a second-order polynomial (see Figure 4(d)). Finally, the scan is warped such that the curve becomes a line as presented in Figures 4(e) and 4(f).

#### 2.1.3. Slice Alignment

The flattening correction does not enforce an alignment through the OCT volume. Thus, in addition to the flattening correction, the warped curves of each B-scan are positioned at the same altitude in the *z*-axis.

### 2.2. Feature Detection

In this research, we choose to detect simple and efficient LBP texture features with regard to each OCT slice and volume. LBP is a texture descriptor based on the signs of the differences of a central pixel with respect to its neighboring pixels [17]. These differences are encoded in terms of binary patterns as follows:

(1) LBPP,R=∑p=0P−1sgp−gc2p,sx=1if  x≥00otherwise,

where *g*_*c*_, *g*_*p*_ are the intensities of the central pixel and a given neighbor pixel, respectively, and *P* is the number of sampling points in the circle of radius *R*.

Ojala et al. further extended the original LBP formulation to achieve rotation invariance at the expense of limiting the texture description to the notion of circular “uniformity” [17]. Referring to the coordinate system defined in Figure 3(a), the LBP codes are computed on each (*x*-*z*) slice, leading to a set of LBP maps, a map for each (*x*-*z*) slice.

Volume encoding is later proposed by Zhao et al. by computing LBP descriptors in three orthogonal planes, so-called LBP-TOP [18]. More precisely, the LBP codes are computed considering the (*x*-*z*) plane, (*x*-*y*) plane, and (*y*-*z*) plane, independently. Thus, three sets of LBP maps are obtained, one for each orthogonal plane.

In this work, we consider rotation invariant and uniform LBP and LBP-TOP features with various sampling points (i.e., {8,16,24}) with respect to different radius (i.e., {1,2, 3}). The number of patterns (LBP_#pat_) in regard to each configuration is reported in Table 2.

### 2.3. Mapping

The mapping stage is used to partition the previously computed LBP maps; for this work, two mapping strategies are defined: (i)* global* and (ii)* local* mapping. The size of the feature descriptor is summarized in Table 3.

#### 2.3.1. Global

**Figure 5:**
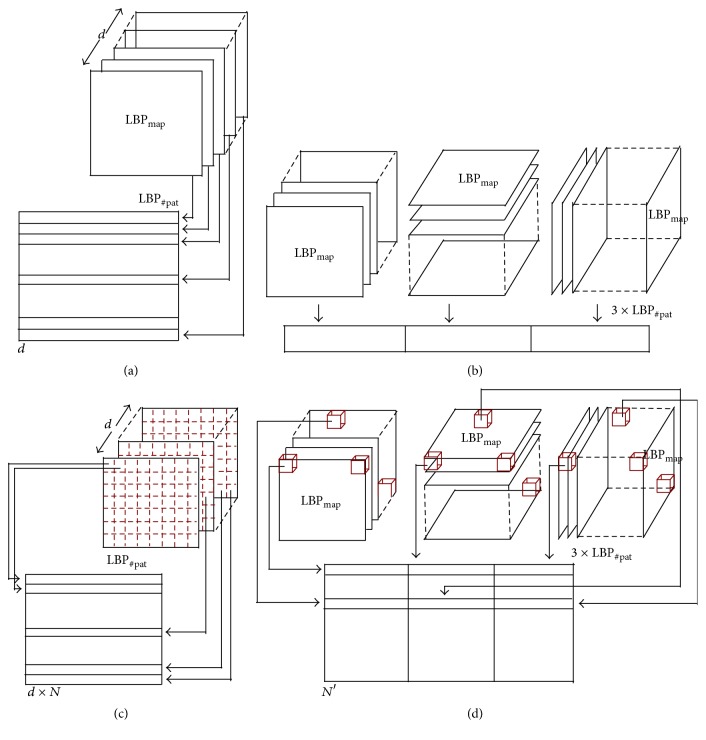
Graphical representation of the feature extraction: (a) extraction of LBP for global mapping, (b) extraction of LBP-TOP for global mapping, (c) extraction of LBP for local mapping, and (d) extraction of LBP-TOP for local mapping.

Global mapping extracts the final descriptors from the 2D feature image for LBP and 3D volume for LBP-TOP. Therefore, for a volume with *d* slices, the* global*-LBP mapping will lead to the extraction of *d* elements, while the* global*-LBP-TOP represents the whole volume as a single element. The* global* mapping for 2D images and 3D volume is shown in Figures 5(a) and 5(b).

#### 2.3.2. Local

Local mapping extracts the final descriptors from a set of (*m* × *m*) 2D patches for LBP and a set of (*m* × *m* × *m*) subvolumes for LBP-TOP. Given *N* and *N*′ as the total number of 2D patches and 3D subvolumes, respectively, the* local*-LBP approach provides *N* × *d* elements, while* local*-LBP-TOP provides *N*′ elements. This mapping is illustrated in Figures 5(c) and 5(d).

### 2.4. Feature Representation

Two strategies are used to describe each OCT volume's texture.

#### 2.4.1. Low-Level Representation

The texture descriptor of an OCT volume is defined as the concatenation of the LBP histograms with the* global* mapping. The LBP histograms are extracted from the previously computed LBP maps (see Section 2.2). Therefore, the LBP-TOP final descriptor is computed through the concatenation of the LBP histograms of the three orthogonal planes with the final size of 3 × LBP_#pat_. More precisely, an LBP histogram is computed for each set of LBP maps (*x*-*z*) plane, (*x*-*y*) plane, and (*y*-*z*) plane, respectively. Similarly, the LBP descriptor is defined through concatenation of the LBP histograms per each (*x*-*z*) slice with the final size of *d* × LBP_#pat_.

#### 2.4.2. High-Level Representation

The concatenation of histograms employed in the low-level representation in conjunction with either* global* or* local* mapping can lead to a high-dimensional feature space. For instance,* local* mapping results in a size of *N* × *d* × LBP_#pat_ for the final LBP descriptor and *N*′ × LBP_#pat_ for the final LBP-TOP descriptor, where *N* and *N*′ are the total number of 2D patches and 3D subvolumes, respectively. High-level representation simplifies this high-dimensional feature space into a more discriminant lower space. BoW approach is used for this purpose [13]. This model represents the features by creating a codebook or visual dictionary, from the set of low-level features. The set of low-level features are clustered using *k*-means to create the codebook with *k* clusters or visual words. After creating the codebook from the training set, the low-level descriptors are replaced by their closest word within the codebook. The final descriptor is a histogram of size *k* which represents the codebook occurrences for a given mapping.

### 2.5. Classification

The last step of our framework consists in the classification of SD-OCT volumes as normal or DME. For that matter, five different classifiers are used: (i) *k*-Nearest Neighbor (NN), (ii) Logistic Regression (LR) [19], (iii) Random Forest (RF) [20], (iv) Gradient Boosting (GB) [21, 22], and (v) Support Vector Machines (SVM) [23, 24]. Details regarding the parameters used in our experiments are provided in Section 3.

## 3. Experiments

A set of three experiments is designed to test the influence of the different blocks of the proposed framework in comparison to our previous work [12]. These experiments are designed as follows:

- *Experiment *1 evaluates the effects of number of words used in BoW (high-level representation).
- *Experiment *2 evaluates the effects of different preprocessing steps and classifiers on high-level representation.
- *Experiment *3 evaluates the effects of different preprocessing steps and classifiers on low-level representation.

Table 4 reports the experiments which have been carried out in [12] as a baseline and outlines the complementary experimentation here proposed. The reminder of this section details the common configuration parameters across the experiments, while the detailed explanations are presented in the following subsections.

All the experiments are performed using a private dataset (see Section 3.1) and are reported as presented in Section 3.2. In all the experiments, LBP and LBP-TOP features are extracted using both* local* and* global* mapping for different sampling points of 8, 16, and 24 for radius of 1, 2, and 3 pixels, respectively. The partitioning for* local*-mapping is set to (7 × 7)-pixel patch for 2D LBP and (7 × 7 × 7)-pixel subvolume for LBP-TOP.

### 3.1. SERI Dataset

This dataset was acquired by the Singapore Eye Research Institute (SERI), using CIRRUS*™* (Carl Zeiss Meditec, Inc., Dublin, CA) SD-OCT device. The dataset consists of 32 OCT volumes (16 DME and 16 normal cases). Each volume contains 128 B-scan with resolution of 512 × 1024 pixels. All SD-OCT images are read and assessed by trained graders and identified as normal or DME cases based on evaluation of retinal thickening, hard exudates, intraretinal cystoid space formation, and subretinal fluid.

### 3.2. Validation

**Figure 6:**
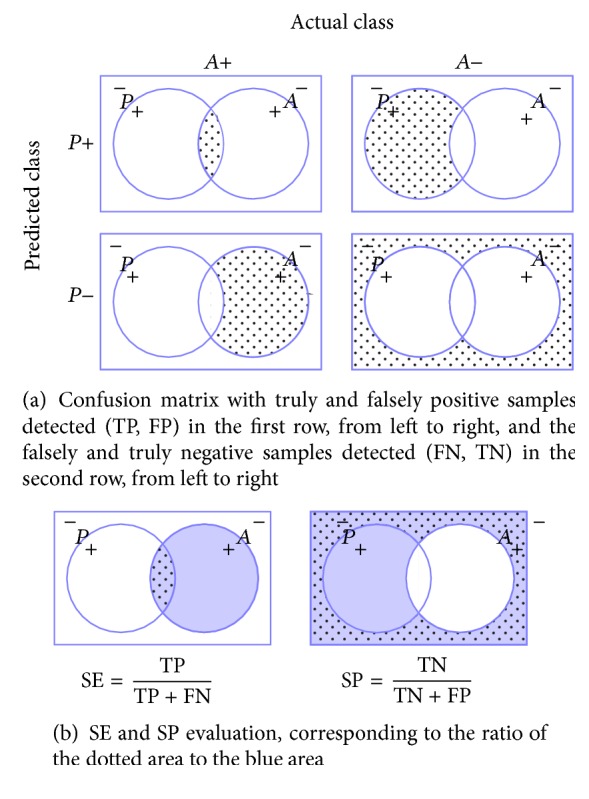
Evaluation metrics: (a) confusion matrix and (b) SE-SP.

All the experiments are evaluated in terms of Sensitivity (SE) and Specificity (SP) using the LOPO-CV strategy, in line with [12]. SE and SP are statistics driven from the confusion matrix as depicted in Figure 6. The SE evaluates the performance of the classifier with respect to the positive class, while the SP evaluates its performance with respect to negative class. The use of LOPO-CV implies that, at each round, a pair of DME-normal volumes is selected for testing while the remaining volumes are used for training. Subsequently, no SE or SP variance can be reported. However, LOPO-CV strategy has been adopted despite this limitation due to the reduced size of the dataset.

### 3.3. Experiment 1

This experiment intends to find the optimal number of words and its effect on the different configurations (i.e., preprocessing and feature representation), on the contrary to [12], where the codebook size was arbitrarily set to *k* = 32.

Several preprocessing strategies are used: (i) NLM, (ii) a combination of NLM and flattening (NLM+F), and (iii) a combination of NLM, flattening, and aligning (NLM+F+A). LBP and LBP-TOP descriptors are detected using the default configuration. Volumes are represented using BoW, where the codebook size ranges within *k* ∈ {10,20,30,…, 100,200,…, 500, 1000}. Finally, the volumes are classified using LR. The choice of this linear classifier avoids the case that the results get boosted by the classifier. In this manner, any improvement would be linked to the preprocessing and the size of the codebook.

The usual build of the codebook consists of clustering the samples in the feature space using *k*-means (see Section 2.4). However, this operation is rather computationally expensive and the convergence of the *k*-means algorithm for all codebook sizes is not granted. Nonetheless, Nowak et al. [25] pointed out that randomly generated codebooks can be used at the expense of accuracy. Thus, the codebook is randomly generated since the final aim is to assess the influence of the codebook size and not the performance of the framework. For this experiment, the codebook building is carried out using random initialization using *k*-means++ algorithm [26], which is usually used as a *k*-means initialization algorithm.

For this experiment, SE and SP are complemented with ACC and F1 score (see (2)). ACC offers an overall sense of the classifier performance, and F1 illustrates the trade-off between SE and precision. Precision or positive predictive value is a measure of algorithm exactness and is defined as a ratio of True Positive over the total predicted positive samples:

(2) ACC=TP+TNTP+TN+FP+FN,F1=2TP2TP+FP+FN.

Table 6 in Appendix shows the results obtained for the optimal dictionary size while the complete set of all ACC and F1 graphics can be found at [27]. According to the obtained results, it is observed that the optimum number of words is smaller for* local*-LBP features in comparison to* local*-LBP-TOP and* global*-LBP, respectively. Using LR classifier, the best performances were achieved using* local*-LBP with 70 words (SE and SP of 75.0%) and* local*-LBP-TOP with 500 words (SE and SP of 75.0% as well). These results are shown in bold in Table 6 in Appendix.

### 3.4. Experiment 2

This experiment explores the improvement associated with (i) different preprocessing methods and (ii) using larger range of classifiers (i.e., linear and nonlinear) on the high-level representation.

All the preprocessing stages are evaluated (NLM, NLM+F, and NLM+F+A). In this experiment, the codebooks for the BoW representation of LBP and LBP-TOP features are computed using regular *k*-means algorithm which is initialized using *k*-means++, where *k* is chosen according to the findings of* Experiment *1. Finally, the volumes are classified using *k*-NN, RF, GB, and SVM. The *k*-NN classifier is used in conjunction with the 3 nearest neighbors rule to classify the test set. The RF and GB classifiers are trained using 100 unpruned trees, while SVM classifier is trained using an RBF kernel and its parameters *C* and *γ* are optimized through grid-search.

Complete list of the obtained results from this experiment is shown in Table 7 in Appendix. Despite the fact that highest performances are achieved when NLM+F or NLM+F+A is used, most configurations decline when applied with extra preprocessing stages. The best results are achieved using SVM followed by RF.

### 3.5. Experiment 3

This experiment replicates* Experiment *2 for the case of low-level representation of LBP and LBP-TOP features extracted using* global* mapping.

The obtained results from this experiment are listed in Table 8 in Appendix. In this experiment, flattening the B-scan boosts the results of the best performing configuration. However, its effects is not consistent across all the configurations. RF has a better performance by achieving better SE (81.2%, 75.0%, and 68.7%), while SVM achieves the highest SP (93.7%), see Table 8 in the Appendix.

In terms of classifier, RF has a better performance than the others despite the fact that the highest SP is achieved using SVM.

## 4. Results and Discussion

Table 5 combines the obtained results from Section 3 with those reported by Lemaitre et al. [12], while detailing the frameworks configurations. This table shows the achieved performances with SE higher than 55%.

The obtained results indicate that expansion and tuning of our previous framework improve the results. Tuning the codebook size, based on the finding of* Experiment *1, leads to an improvement of 6% in terms of SE (see Table 5 at lines 7 and 13). Furthermore, the fine-tuning of our framework (see Section 2) also leads to an improvement of 6% in both SE and SP (see Table 5 at lines 1 and 13). Our framework also outperforms the proposed method of [10] with an improvement of 20% and 36% in terms of SE and SP, respectively.

Note that although the effects of preprocessing are not consistent through all the performances, the best results are achieved with NLM+F and NLM+F+A configurations as preprocessing stages. In general, the configurations presented in* Experiment *2 outperform the others, in particular the high-level representation of locally mapped features with an SVM classifier. Focusing on the most desirable radius and sampling point configuration, smaller radius and sampling points are more effective in conjunction with local mapping, while global mapping benefits from larger radius and sampling points.

## 5. Conclusions

The work presented here addresses automatic classification of SD-OCT volumes as normal or DME. In this regard, an extensive study is carried out covering the (i) effects of different preprocessing steps, (ii) influence of different mapping and feature extraction strategies, (iii) impact of the codebook size in BoW, and (iv) comparison of different classification strategies.

While outperforming the previous studies [10, 12], the obtained results in this research showed the impact and importance of optimal codebook size, the potential of 3D features, and high-level representation of 2D features while extracting from local patches.

The strengths of SVM while being used along with BoW approach and RF classifier while being used with global mapping were shown. In terms of preprocessing steps, although the highest performances are achieved while alignment and flattening were used in the preprocessing, it was shown that the effects of these extra steps are not consistent for all the cases and do not guarantee a better performance.

Several avenues for future directions can be explored. The flattening method proposed by Liu et al. flattens roughly the RPE due to the fact that the RPE is not segmented. Thus, in order to have a more accurate flattening preprocessing, the RPE layer should be presegmented as proposed by Garvin et al. [28]. In this work, the LBP invariant to rotation was used and the number of patterns encoded is reduced. Once the data are flattened, the nonrotation invariant LBP could be studied since this descriptor encodes more patterns. In addition to LBP, other feature descriptors can be included in the framework.

## Figures and Tables

**Table 1:** Summary of the state-of-the-art methods.

Reference	Diseases			Data size	Preprocessing				Features	Representation	Classifier	Evaluation	Results
	AMD	DME	Normal		Denoise	Flatten	Aligning	Cropping					
[9]	✓	✓	✓	45	✓	✓		✓	HOG		Linear SVM	ACC	86.7%, 100%, and 100%
[10]	✓		✓	384					Texton	BoW, PCA	RF	AUC	0.984
[11]	✓	✓	✓	326		✓	✓		Edge, LBP	PCA	SVM-RBF	AUC	0.93
[12]		✓	✓	62	✓				LBP-LBP-TOP	PCA, BoW, and histogram	RF	SE, SP	87.5%, 75%

**Table 2:** Number of patterns (LBP_#pat_) for different sampling points and radius ({*P*, *R*}) of the LBP descriptor.

Sampling point for a radius ({*P*, *R*})			
	{8, 1}	{16, 2}	{24, 3}
LBP_#pat_	10	18	26

**Table 3:** Size of a descriptor for an SD-OCT volume. *d* denotes the number of slices in the volume, *N* the number of 2D windows, and *N*′ the number of 3D subvolumes, respectively.

	Global mapping	Local mapping
LBP	*d* × LBP_#pat_	(*N* × *d*) × LBP_#pat_
LBP-TOP	1 × (3 × LBP_#pat_)	*N*′ × (3 × LBP_#pat_)

**Table 4:** The outline and summary of the performed experiments.

Common	Dataset	Preprocessing	Features	Mapping	Representation	Classification	Evaluation
	SERI	NLM	LBP, LBP-TOP *P* = {8,16,24} *R* = {1,2, 3}				Leave-One-Patient Out Cross-Validation (LOPO-CV) SE, SP
Baseline [12] Goal: evaluation of features, mapping, and representation	+ Duke	~	~	*Global* *Local*	BoW Histogram	RF	+ comparison with [10]

Experiment 1 Goal: finding the optimum number of words	~	+F +F+A	~	*Global* *Local*	BoW *k* ∈ *K*	LR	+ACC, F1 score (F1)

Experiment 2 Goal: evaluation of different preprocessing and classifiers for high-level features	~	+F +F+A	~	*Global* *Local*	BoW Optimal *k*	3-NN RF SVM GB	~

Experiment 3 Goal: evaluation of different preprocessing and classifiers for low-level features	~	+F +F+A	~	*Global*	Histogram	3-NN RF SVM GB	~

~ indicates that common configuration applies.

**Table 5:** Summary of all the results in descending order.

Line	Experiment	Evaluation		Pre-processing	Feat. Detection				Mapping	Feat. Representation	Classifier	BoW
		SE	SP		Type	{8,1}	{16,2}	{24,3}				
1	2	81.2	93.7	NLM+F	LBP	✓			Local	High	SVM	✓
2	2	75.0	93.7	NLM+F+A	LBP	✓			Local	High	SVM	✓
3	2	75.0	93.7	NLM	LBP	✓			Local	High	SVM	✓
4	2	75.0	100	NLM	LBP-TOP		✓		Local	High	SVM	✓
5	2	81.2	87.5	NLM	LBP-TOP	✓			Local	High	SVM	✓
6	2	81.2	87.5	NLM+F+A	LBP-TOP		✓		Local	High	RF	✓
7	2	81.2	81.2	NLM	LBP	✓			Local	High	RF	✓
8	3	81.2	81.2	NLM	LBP-TOP			✓	Global	Low	RF	
9	2	81.2	81.2	NLM+F	LBP-TOP	✓			Local	High	SVM	✓
10	3	81.2	81.2	NLM+F+A	LBP-TOP			✓	Global	Low	GB	
11	3	81.2	81.2	NLM+F	LBP-TOP			✓	Global	Low	RF	
12	2	75.0	87.5	NLM	LBP	✓			Local	High	*k*-NN	✓
13	Lemaitre et al. [12]	75.0	87.5	NLM	LBP	✓			Local	High	RF	✓
14	Lemaitre et al. [12]	75.0	87.5	NLM	LBP-TOP		✓		Global	Low	RF	
15	2	68.7	93.7	NLM	LBP	✓			Global	High	RF	✓
16	3	75	81.2	NLM+F+A	LBP-TOP			✓	Global	Low	RF	
17	2	68.7	81.2	NLM	LBP-TOP		✓		Local	High	RF	✓
18	3	62.5	93.7	NLM	LBP-TOP		✓		Global	Low	SVM	
19	3	68.7	87.5	NLM	LBP-TOP		✓		Global	Low	RF	
20	3	68.7	81.2	NLM	LBP-TOP				Global	Low	RF	
21	3	75.0	75.0	NLM	LBP-TOP				Global	Low	RF	
22	3	68.7	75.0	NLM+F	LBP-TOP	✓			Global	Low	SVM	
23	3	56.2	75.0	NLM	LBP			✓	Global	Low	RF	
24	3	56.2	75.0	NLM+F	LBP		✓		Global	Low	*k*-NN	
25	3	56.2	75.0	NLM+F+A	LBP		✓		Global	Low	*k*-NN	
26	Venhuizen et al. [10]	61.5	58.8									

**Table 6:** Experiment 1—optimum number of words for each configuration as a result of LR classification, for high-level feature extraction of *global* and *local*-LBP, and *local*-LBP-TOP features with different preprocessing. The preprocessing includes NF, F, and F+A. The achieved performance is indicated in terms of ACC, F1, SE, and SP.

Features	Preprocessing	{8,1}					{16,2}					{24,3}				
		ACC%	F1%	SE%	SP%	Number of words	ACC%	F1%	SE%	SP%	Number of words	ACC%	F1%	SE%	SP%	Number of words
*Global*-LBP	NF	81.2	78.5	68.7	93.7	500	62.5	58.0	56.2	62.5	80	62.5	62.5	62.5	62.5	80
	F	71.9	71.0	68.7	75.0	400	68.7	66.7	62.5	75.0	300	68.7	66.7	62.5	75.0	300
	F+A	71.9	71.0	68.7	75.0	500	71.9	71.0	68.7	75.0	200	75.0	68.7	68.7	68.7	500

*Local*-LBP	NF	**75.0**	**75.0**	**75.0**	**75.0**	**70**	65.6	64.5	62.5	68.7	90	62.5	60.0	56.2	68.7	30
	F	75.0	73.3	68.7	81.2	30	71.8	61.0	68.7	75.0	70	62.5	62.5	62.5	62.5	100
	F+A	75.0	69.0	62.5	81.2	40	71.9	71.0	68.7	75.0	200	68.7	66.7	68.7	62.5	10

*Local*-LBP-TOP	NF	68.7	68.7	68.7	68.7	400	**75.0**	**75.0**	**75.0**	**75.0**	**500**	71.9	71.0	68.7	75.0	60
	F	68.7	68.7	68.7	68.7	300	68.7	66.7	62.5	75.0	50	75.0	76.5	81.2	68.7	80
	F+A	75.0	73.3	68.7	81.2	100	75.0	73.3	68.7	81.2	90	75.0	69.0	62.5	81.2	70

**(a):** 

Features	Preprocessing	*k*-NN						SVM					
		{8,1}		{16,2}		{24,3}		{8,1}		{16,2}		{24,3}	
		SE%	SP%	SE%	SP%	SE%	SP%	SE%	SP%	SE%	SP%	SE%	SP%
*Global*-LBP	NF	43.7	93.7	43.7	87.5	43.7	62.5	68.7	87.5	62.5	62.5	50.0	56.2
	F	43.7	56.2	50.0	75.0	62.5	56.2	56.2	56.2	56.2	75.0	56.2	68.7
	FA	56.2	62.5	43.7	81.2	68.7	56.2	56.2	68.7	68.7	68.7	56.2	75.0

*Local*-LBP	NF	***75.0***	***87.5***	50.0	68.7	43.7	43.7	**75.0**	**93.7**	50.0	75.0	56.2	56.2
	F	*56.2*	*56.2*	50.0	50.0	50.0	43.7	**81.2**	**93.7**	68.7	68.7	68.7	75.0
	FA	*56.2*	*43.7*	50.0	75.0	50.0	62.5	**75.0**	**93.7**	75.0	68.7	68.7	68.7

*Local*-LBP-TOP	NF	56.2	75.0	56.2	75.0	62.5	56.2	***81.2***	***87.5***	***75.0***	***100***	56.2	75.0
	F	62.5	43.7	37.5	68.7	43.7	62.5	***81.2***	***81.2***	*75.0*	*68.7*	81.2	68.7
	F+A	56.2	56.2	68.7	50.0	43.7	62.5	*62.5*	*75.0*	*68.7*	*75.0*	62.5	81.2

**(b):** 

Features	Preprocessing	RF						GB					
		8^riu2^		16^riu2^		24^riu2^		8^riu2^		16^riu2^		24^riu2^	
		SE%	SP%	SE%	SP%	SE%	SP%	SE%	SP%	SE%	SP%	SE%	SP%
*Global*-LBP	NF	***68.7***	***93.7***	43.7	62.5	50.0	68.7	56.2	50.0	37.5	31.2	50.0	43.7
	F	*56.2*	*50.0*	56.2	75.0	50.0	75.0	50.0	56.2	56.2	75.0	43.7	62.5
	FA	*68.7*	*50.0*	56.2	62.5	62.5	56.2	56.2	50.0	68.7	50.0	43.7	75.0

*Local*-LBP	NF	***81.2***	***81.2***	62.5	56.2	56.2	56.2	75.0	62.5	68.7	87.5	50.0	75.0
	F	*56.2*	*81.2*	62.5	68.7	68.7	62.5	68.7	75.0	50.0	75.0	50.0	62.5
	FA	*68.7*	*62.5*	62.6	68.7	43.7	43.7	56.2	50.0	68.7	56.2	50.0	50.0

*Local*-LBP-TOP	NF	68.7	62.5	**68.7**	**81.2**	68.7	68.7	37.5	68.7	62.5	81.2	62.5	50.0
	F	50.0	62.5	62.5	62.5	43.7	75.0	50.0	56.2	43.7	62.5	50.0	62.5
	F+A	50.0	62.5	**81.2**	**87.5**	50.0	68.7	56.2	62.5	81.2	68.7	75.0	68.7

**Table:** (a)

Features	Preprocessing	*k*-NN						SVM					
		{8,1}		{16,2}		{24,3}		{8,1}		{16,2}		{24,3}	
		SE%	SP%	SE%	SP%	SE%	SP%	SE%	SP%	SE%	SP%	SE%	SP%
*Global*-LBP	NF	37.5	50.0	25.0	50.0	37.5	68.7	56.2	62.5	56.2	43.7	56.2	68.7
	F	62.5	50.0	56.2	75.0	62.5	68.7	75.0	68.7	62.5	62.5	62.5	68.7
	FA	56.2	50.0	56.2	75.0	62.5	68.7	75.0	68.7	62.5	62.5	62.5	68.7

*Global*-LBP-TOP	NF	31.2	93.7	37.5	100.0	37.5	81.2	62.5	75.0	***62.5***	***93.7***	56.2	87.5
	F	50.0	56.2	56.2	75.0	56.2	62.5	68.7	75.0	*43.7*	*68.7*	68.7	56.2
	F+A	75.0	43.7	56.2	43.7	68.7	50.0	68.7	62.5	*62.5*	*56.2*	56.2	68.7

**Table:** (b)

Features	Preprocessing	RF						GB					
		8^riu2^		16^riu2^		24^riu2^		8^riu2^		16^riu2^		24^riu2^	
		SE%	SP%	SE%	SP%	SE%	SP%	SE%	SP%	SE%	SP%	SE%	SP%
*Global*-LBP	NF	43.7	62.5	43.7	62.5	56.2	75	43.7	43.7	43.7	37.5	37.5	31.25
	F	56.2	56.2	68.7	62.5	62.5	68.7	25	56.2	50.0	43.7	25.0	43.7
	F+A	65.2	56.2	50.0	50.0	56.2	68.7	43.75	62.5	62.5	50.0	31.2	31.2

*Global*-LBP-TOP	NF	56.2	68.7	***68.7***	***87.5***	**68.7**	**81.2**	68.7	68.7	75.0	50.0	*56.2*	*43.7*
	F	56.2	62.5	*81.2*	*68.7*	**81.2**	**81.2**	56.2	62.5	62.5	68.7	*68.7*	*81.2*
	F+A	68.7	62.5	*75.0*	*68.7*	**75.0**	**81.2**	56.2	43.7	62.5	62.5	***75.0***	***75.0***

## Appendix

### Complementary Results for Experiments 1, 2, and 3

See Tables 6, 7, and 8.
